# Length of Stay in ED for Patients Awaiting Inpatient Psychiatric Beds

**DOI:** 10.1192/bjo.2025.10425

**Published:** 2025-06-20

**Authors:** Anusha Prabhu, Chun Chiang Sin Fai Lam

**Affiliations:** King’s College Hospital, London, United Kingdom

## Abstract

**Aims:** The King’s Fund have noted that the number of people waiting more than 12 hours after decision to admit to admission has increased significantly over the last ten years. With increasing demand for psychiatric inpatient beds, waiting times for mental health patient admissions are projected to similarly increase. This study aimed to investigate the length of stay for patients within a large inner city emergency department (ED) awaiting a mental health inpatient admission.

**Methods:** Time of decision to admit (DTA) and time of discharge were recorded and reviewed for all patients awaiting a psychiatric inpatient admission in August 2024.

**Results:** In total 101 patients were assessed for a psychiatric admission during this period. The average length of stay from arrival to discharge from the ED was 1 day and 7 hours, with the longest length of stay being 5 days and 5 hours. The average time from DTA to discharge from ED was 1 day and 16 minutes with 73.3% of all patients spending more than 12 hours waiting for their bed from DTA. It is noted, that out of the 101 patients initially assessed for admission, 14 were discharged to the community.

**Conclusion:** Emergency departments are less likely to be equipped with the resources, physical infrastructure and trained staff required for caring for patients with high acuity of mental illness for prolonged periods of time. This study provides information demonstrating the strain that services have in providing the right care in the right place. With the reduction in available inpatient psychiatric beds by 24% since 2010, ongoing consideration needs to be given on how appropriate care can be delivered to this cohort of vulnerable and unwell patients.

